# Protective effects of nano-zinc and nano-selenium compared with a toxin binder in broiler chickens fed aflatoxin B1-contaminated diets through antioxidant, immune, and skeletal responses

**DOI:** 10.1016/j.vas.2026.100888

**Published:** 2026-10-06

**Authors:** Fouad Kadhim Mohsin, Kamran Taherpour, Hossein Ali Ghasemi, Hassan Shirzadi, Fatemeh Tavakolinasab

**Affiliations:** a Department of Animal Science, Faculty of Agriculture and Natural Resources, Ilam University, Pajouhesh Blvd, Ilam, Ilam, 6939177111, Iran; b Department of Animal Science, Faculty of Agriculture and Environment, Arak University, Shahid Beheshti Street, Arak, Markazi Province, 38156-8-8349, Iran

**Keywords:** Aflatoxin B1, Broiler, Nano-zinc, Nano-selenium, Antioxidant status

## Abstract

Aflatoxin B1 (AFB1) can impair broiler performance, redox balance, and skeletal health. This 42-day study evaluated whether partial replacement of conventional inorganic zinc (Zn) and selenium (Se) sources with nanoforms could mitigate AFB1-associated responses and compared this source-substitution strategy with the use of commercial toxin binder. A total of 792 day-old male Ross 308 broilers were allocated to six treatments (6 replicate pens/treatment, 22 birds/pen; pen served as the experimental unit): Control; AF (0.5 mg AFB1/kg); AF+TB (2 g toxin binder/kg); AF+NZ (40 mg Zn/kg supplied as nano-zinc in place of an equivalent amount from zinc sulfate); AF+NS (0.1 mg Se/kg supplied as nano-selenium in place of an equivalent amount from sodium selenite); and AF+NZ+NS (combined nano-Zn and nano-Se partial replacement). Supplemental Zn and Se were maintained at 120 and 0.3 mg/kg, respectively. Relative to AF, AF+TB and AF+NZ+NS increased body weight gain by 9.7% and 6.3%, feed efficiency by 6.8% and 5.7%, total antioxidant capacity by 37.6% and 34.5%, and serum GSH by 38.6% and 35.7%, respectively, while reducing serum MDA by 31.0% and 32.1% (P < 0.05). Both treatments also improved selected immunoglobulin measures, hepatic expression of NFE2L2, GPX1, and SOD1, culture-based bacterial counts, tibial length and tibia ash content, although AF+NZ+NS did not consistently outperform the single nano-mineral treatments and did not fully restore the control phenotype. Under the conditions of this 42-day, 0.5 mg/kg AFB1 challenge, combined partial replacement with nano-Zn and nano-Se improved several measured responses, including productive performance, antioxidant status, and tibia quality.

## 1 Introduction

Aflatoxin B1 (AFB1), produced mainly by Aspergillus flavus and Aspergillus parasiticus, is a major feedborne threat to broiler health, performance, and food safety. Contemporary feed surveillance continues to document frequent contamination of feed with AFB1 and its co-occurrence with other mycotoxins, emphasizing the need for practical prevention and mitigation strategies (Liu et al., 2025a; Sun et al., 2026). In broilers, dietary AFB1 can depress feed intake, weight gain, feed efficiency, carcass yield, and nutrient utilization. Recent work has also confirmed the accumulation of AFB1 residues in the liver, together with oxidative and intestinal injury during experimental exposure (Hou et al., 2022; Lai et al., 2022; Choi et al., 2025; Rao et al., 2026). Its toxicity is largely mediated by hepatic bioactivation of AFB1 to reactive metabolites, followed by lipid peroxidation, glutathione depletion, mitochondrial dysfunction, and dysregulation of *NFE2L2*- and *NF-κB*-related signaling, which collectively promote oxidative stress, inflammation, and immune suppression (Guo et al., 2021; Li et al., 2022; Moloi et al., 2024). The intestine and skeletal system are also recognized AFB1 targets (Tavangar et al., 2021; Sarker et al., 2023; Paneru et al., 2024).

Commercial toxin binders are commonly used to limit gastrointestinal AFB1 availability, although their efficacy depends on binder composition, dose, binding affinity, gut conditions, and potential interactions with nutrients (Rashidi et al., 2020; Saleh et al., 2024; Cuccato et al., 2025). Nutritional strategies that strengthen host antioxidant and immune defenses may therefore provide a complementary approach. Zinc supports enzymatic activity, protein synthesis, metallothionein-mediated protection, epithelial repair, and immune-cell function (De Grande et al., 2020; Nasiri et al., 2025), whereas selenium is required for the activity of glutathione peroxidases and thioredoxin reductases involved in peroxide detoxification and immune regulation (Dalia et al., 2020; Liu et al., 2025b). In chicks, nano-selenium has also been experimentally associated with reduced ROS/AP-1 signaling and CYP1A1/CYP2A6-mediated AFB1 bioactivation (Deng et al., 2024). Recent nanoparticle-based interventions provide additional evidence of biological activity in poultry challenge models: nano-chitosan partially mitigated combined AFB1/fumonisin toxicity (Khashan et al., 2025), and zinc oxide nanoparticles showed antibacterial activity in broilers challenged with colibacillosis (Bahadur et al., 2024). More broadly, the antimicrobial activity of nanoparticles is strongly influenced by particle size, surface properties, concentration, and biological context (Usman et al., 2025). These same properties can also increase reactivity and potential toxicological risk; consequently, responses to nano-minerals should be interpreted as dependent on source, dose, and particle characteristics rather than as evidence of universal superiority (Al-Jaf and Rashid, 2025; Piray et al., 2025; Yang et al., 2025).

Despite these advances, studies directly comparing nano-zinc and nano-selenium, administered alone or in combination, with a commercial toxin binder under the same AFB1 challenge remain scarce. The treatment structure used in the present study was designed to address this unresolved question while separating mineral-source substitution from simple dose escalation. Specifically, nano sources supplied 40 mg Zn/kg and 0.1 mg Se/kg, each representing one-third of the respective supplemental Zn (120 mg/kg) and Se (0.3 mg/kg) levels and replacing equivalent amounts of zinc sulfate or sodium selenite rather than increasing total mineral supplementation. Partial rather than complete replacement retained a conventional inorganic fraction and provided a conservative comparison of mineral-source substitution; the study was not designed as a nano-mineral dose-response or complete-replacement trial. We hypothesized a priori that combined nano-Zn and nano-Se would provide a broader and more consistent pattern of protection than either nano-mineral alone and could approach the response achieved with the toxin binder across growth performance, carcass traits, redox status, immune indices, selected culture-based bacterial counts, and tibial characteristics.

## 2 Materials and methods

### 2.1 Animal ethics

All procedures involving bird care, management, and sample collection were approved by the Animal Ethics Committee of Ilam University, Iran (approval number: IR.ILAM.REC.1405.003). All experiments were performed in accordance with relevant institutional guidelines and regulations, and the study was reported in accordance with the ARRIVE guidelines.

### 2.2 Birds, management, and experimental design

A total of 792 day-old male Ross 308 broiler chicks with similar initial body weights were obtained from a single flock and used in a 42-day feeding trial. Birds were randomly assigned to six dietary treatments, with six replicate pens per treatment and 22 birds per replicate pen. Chicks were housed in floor pens measuring 1.5 × 1.8 m under controlled environmental conditions. Feed and water were provided ad libitum throughout the experiment. The house temperature was maintained at approximately 34°C during the first week and was gradually reduced to approximately 23°C during the subsequent weeks. Adequate ventilation and lighting were provided throughout the rearing period. The lighting program consisted of 24 h of light during the first 3 days, followed by 23 h of light and 1 h of darkness from day 4 to day 42. The poultry house was regularly cleaned and disinfected to maintain hygienic conditions. The replicate pen was considered the experimental unit for treatment allocation and performance analyses. For outcomes measured in two birds per pen, the mean value for the two birds was used as the pen-level observation. The feeding trial commenced immediately after placement of the day-old chicks; therefore, no separate pre-experimental acclimatization period was used.

### 2.3 Experimental diets and treatments

All diets were formulated to meet standard nutrient requirements for broiler chickens. Table 1 presents the ingredient composition and calculated nutrient values of the starter, grower, and finisher basal diets; whereas Table 2 separately reports the analyzed Zn and Se concentrations. The same basal formulation was used across treatments within each feeding phase, and diets were formulated to be nutritionally equivalent apart from the intended AFB1 challenge, toxin-binder inclusion, and/or substitution of portions of the supplemental inorganic Zn and Se sources with their respective nano sources. The six experimental treatments were as follows: Control, basal diet without AFB1 or additional treatment supplementation; AF, basal diet contaminated with AFB1 at 0.5 mg/kg; AF+TB, AFB1-contaminated diet supplemented with a commercial toxin binder at 2 g/kg; AF+NZ, AFB1-contaminated diet in which 40 mg Zn/kg of diet was supplied as nano-zinc, replacing an equivalent amount of supplemental Zn from zinc sulfate; AF+NS, AFB1-contaminated diet in which 0.1 mg Se/kg of diet was supplied as nano-selenium, replacing an equivalent amount of supplemental Se from sodium selenite; and AF+NZ+NS, AFB1-contaminated diet containing both partial nano-mineral replacements.

**Table 1:** Ingredient composition and calculated nutrient values of starter, grower, and finisher basal diets on an as-fed basis (g/kg, unless otherwise stated).

Items	**Starter (d 0 to 10)**	**Grower (d 11 to 24)**	**Finisher (d 25 to 42)**
Corn grain	545.1	601.7	642.7
Soybean meal, 44%	331.4	256.9	220.5
Corn gluten meal, 60%	45.1	66.5	55.2
Soybean oil	25.0	25.0	35.0
Monocalcium phosphate	14.8	13.3	11.8
Limestone	15.8	14.6	13.5
Salt (NaCl)	2.5	1.7	1.6
Sodium bicarbonate	0.6	1.8	1.9
Vitamin premix^1^	2.5	2.5	2.5
Mineral premix^2^	2.5	2.5	2.5
L-Lysine.HCL	4.3	4.5	4.1
DL-Methionine	2.8	2.2	2.1
L-Threonine	2.0	1.6	1.4
L-Valine	0.6	0.2	0.2
Filler 3	5	5	5
Total	1000	1000	1000
Calculated nutritive value			
Metabolizable energy, kcal/kg	3000	3100	3200
Crude protein	230.0	215.0	175.5
Crude fat	49.6	51.7	63.4
Calcium	9.6	8.7	7.9
Available phosphorus	4.8	4.4	4.0
Digestible lysine	12.8	11.5	10.3
Digestible methionine	6.2	5.5	5.4
Digestible TSAA^4^	9.5	8.7	8.0
Digestible threonine	8.6	7.7	6.9
Digestible valine	9.6	8.7	7.8
Digestible tryptophan	1.9	1.7	1.5
Digestible arginine	12.7	11.2	10.1
Digestible isoleucine	8.2	7.5	6.8
Digestible leucine	13.7	12.3	11.0
Sodium	1.6	1.6	1.6
Potassium	9.5	8.1	7.4
Chlorine	2.8	2.4	2.2
DEB^5^, mEq/100 g	250	230	215

1 Supplied per kg diet: 18 mg retinol, 4 mg cholecalciferol, 36 mg a-tocopherol acetate, 2 mg vitamin K_3_, 1.75 mg vitamin B_1_, 6.6 mg vitamin B_2_, 9.8 mg niacin, 29.65 mg pantothenic acid, 2.94 mg vitamin B_6_, 1 mg folic acid, 0.015 mg vitamin B_12_, 0.1 mg biotin, 250 mg choline chloride and 1 mg ethoxyquin.

2 Supply of mineral elements, with the exception of zinc and selenium (which was included at different levels according to treatment), was the same across all diets, and the following amounts were provided per kilogram of diet: Mn (manganese oxide), 120 mg; Fe (ferrous sulfate), 20 mg; Cu (cupric sulfate), 16 mg; I (potassium iodide), 1.25 mg

3 Building sand served solely as an inert, nonnutritive filler. Its inclusion was adjusted to maintain equal diet mass when treatment-specific mineral premixes were incorporated; no nutritive contribution was assigned to the filler.

4 Total sulfur amino acids

5 DEB (dietary electrolyte balance) = (Na^+^, mEq/kg + K^+^, mEq/kg) – CL^−^, mEq/kg.

**Table 2:** Supplemental levels and analyzed contents of zinc and selenium in experimental diets (mg/kg).

Item	Basal diet (without supplemental Zn and Se)	Basal diet (with supplemental Zn and Se)^1^	AF+NZ	AF+NS	AF+NZ+NS
Supplemental level					
Zinc (from zinc sulfate)	-	120	80	120	80
Zinc (from nano source)	-	-	40	-	40
Selenium (from sodium selenite)	-	0.3	0.3	0.2	0.2
Selenium (from nano source)	-	-	-	0.1	0.1
Analyzed mineral content					
Starter (d 1 to 10)					
Zinc	32	151	153	150	154
Selenium	0.102	0.40	0.42	0.39	0.41
Grower (d 11 to 24)					
Zinc	30	147	149	146	150
Selenium	0.098	0.41	0.40	0.41	0.42
Finisher (d 25 to 42)					
Zinc	29	145	149	147	149
Selenium	0.101	0.39	0.40	0.42	0.42

1 The basal-diet column applies to Control, AF, and AF+TB. AF+NZ contained 40 mg/kg supplemental Zn from nano-zinc replacing an equivalent amount from zinc sulfate; AF+NS contained 0.1 mg/kg supplemental Se from nano-selenium replacing an equivalent amount from sodium selenite; AF+NZ+NS contained both partial replacements. Nano-minerals therefore changed mineral source allocation without increasing the supplemental Zn or Se amounts.

In the Control, AF, and AF+TB treatments, supplemental Zn and Se were supplied at 120 mg Zn/kg and 0.3 mg Se/kg of diet, respectively, entirely as zinc sulfate and sodium selenite. In treatments containing nano-zinc, 40 mg Zn/kg of diet (one-third of the supplemental Zn level) was supplied as nano-zinc, replacing an equivalent amount of Zn from zinc sulfate; thus, supplemental Zn remained 120 mg/kg, consisting of 40 mg/kg from nano-zinc and 80 mg/kg from zinc sulfate. In treatments containing nano-selenium, 0.1 mg Se/kg of diet (one-third of the supplemental Se level) was supplied as nano-selenium, replacing an equivalent amount of Se from sodium selenite; thus, supplemental Se remained 0.3 mg/kg, consisting of 0.1 mg/kg from nano-selenium and 0.2 mg/kg from sodium selenite. This one-third replacement scheme was used to test the effects of mineral-source substitution while avoiding additional Zn or Se supplementation. Supplemental Zn and Se levels and analyzed total-diet Zn and Se concentrations are presented in Table 2.

### 2.4 Exposure characterization

Dietary AFB1 contamination was generated by culturing *Aspergillus flavus* in yeast extract medium and subsequently transferring the fungal culture to Iranian rice (Hashemi variety), as previously described (Mohammadi et al., 2024, 2025). The rice was inoculated with 500 mL of a fungal suspension containing 10⁸ spores/mL and incubated at 28°C for 7 days. Following incubation, the contaminated rice was autoclaved, dried, ground to a fine powder, and analyzed for AFB1 using high-performance liquid chromatography (HPLC) with fluorescence detection. The contaminated rice contained 41.3 mg AFB1/kg; therefore, approximately 12.1 g of contaminated rice per kilogram of diet was incorporated by replacing an equivalent amount of corn to achieve a nominal AFB1 concentration of 0.5 mg/kg. Representative composite samples of each starter, grower, and finisher diet were analyzed using immunoaffinity-column cleanup followed by HPLC with fluorescence detection. The dietary AFB1 concentration of 0.5 mg/kg, administered for 42 days, was used as an experimental challenge level.

### 2.5 Characteristics of supplements used

A commercial toxin binder, Agrabond (Agranco Corp. Co., FL, USA), was included in the AF+TB treatment at 2 g/kg of diet. Agrabond is a calcium- and sodium-based aluminosilicate adsorbent with cation-exchange properties (Rashidi et al., 2020). According to the manufacturer, the product has a minimum aflatoxin adsorption efficacy of approximately 93%. Nano-zinc particles were obtained from Sigma-Aldrich, Inc. (Merck KGaA, Darmstadt, Germany). According to the supplier specifications, the nano-zinc particles had a diameter of <50 nm and a purity of >97%. Nano-selenium particles were obtained from American Elements (Los Angeles, CA, USA), with a supplier-reported size range of 10–45 nm and a purity of 99%. In the respective experimental diets, nano-zinc and nano-selenium supplied 40 mg Zn/kg and 0.1 mg Se/kg of diet, respectively, as partial replacements for equivalent amounts of Zn and Se from the corresponding inorganic sources.

### 2.6 Performance and carcass traits

Broiler performance was evaluated on a pen basis throughout the 42-day experimental period. Body weight and feed intake were recorded for each replicate pen, and body weight gain was calculated as the difference between final and initial body weight. Feed intake was determined after correction for residual feed. Mortality was recorded daily, and performance calculations were adjusted for mortality. Feed efficiency was calculated as the ratio of body weight gain to feed intake over the entire experimental period.

At 42 days of age, two birds from each replicate pen, with body weights close to the mean body weight of their respective pen, were selected for carcass-trait evaluation. Birds were weighed individually to determine pre-slaughter body weight and were then euthanized by cervical dislocation. Thereafter, carcasses were defeathered and eviscerated, and carcass yield was determined. The breast, thigh, drumstick, abdominal fat, liver, heart, spleen, thymus, and bursa of Fabricius were carefully removed and weighed.

Carcass traits and organ weights were expressed as percentages of pre-slaughter body weight using the following formula:

Relativeweight(%)=[weightofcarcass,cut,abdominalfat,ororgan(g)/pre−slaughterbodyweight(g)]×100

### 2.7 Serum antioxidant status and hormone profile

On day 42, the two birds selected from each pen for carcass-trait evaluation were sampled before euthanasia. Blood samples were collected from the wing vein into serum-separation tubes and centrifuged to obtain serum, following the sampling approach used in our previous broiler studies (Mohammadi et al., 2024; Mohammadizad et al., 2025). Serum was separated, aliquoted, and stored at −20°C until biochemical analysis. Serum total antioxidant capacity (TAC), glutathione peroxidase (GPx) activity, superoxide dismutase (SOD) activity, catalase (CAT) activity, malondialdehyde (MDA) concentration, and glutathione (GSH) concentration were determined using commercial spectrophotometric assay kits according to the manufacturers’ instructions. TAC was measured using a commercial assay kit, whereas GPx, SOD, CAT, MDA, and GSH were assayed using the corresponding commercial kits from Cayman Chemical Co. (Ann Arbor, MI, USA). Absorbance was measured using an automated spectrophotometer (Chem 200; Gesan Production Srl, Campobello, Italy), and all assays were conducted according to the manufacturers’ protocols.

The serum hormone profile included corticosterone, triiodothyronine (T3), and thyroxine (T4). Serum T3 and T4 concentrations were determined using enzyme-linked immunosorbent assay kits (Pishtaz Teb, Tehran, Iran; mean inter-assay and intra-assay coefficients of variation: T3, 2.7% and 4.2%, respectively; T4, 3.9% and 4.8%, respectively). Serum corticosterone concentration was quantified using a chicken-specific ELISA kit (ZellBio GmbH, Ulm, Germany).

### 2.8 Serum immunoglobulin concentrations

On days 24 and 42, two birds per replicate pen, with body weights close to the replicate mean, were selected for blood sampling. Blood samples were collected from the wing vein into serum-separation tubes and centrifuged at 2,500 × g for 15 min at 4°C. The separated serum was collected, aliquoted, and stored at −20°C until analysis. The sampling and serum-separation procedures were based on those used in previous broiler studies (Biabani et al., 2024; Ghasemi et al., 2025). Serum immunoglobulin concentrations were determined using enzyme-linked immunosorbent assay (ELISA) kits according to the manufacturers’ instructions and procedures described in previous related studies. Serum IgA, IgG/IgY, and IgM concentrations were quantified using chicken-specific ELISA quantification kits (Bethyl Laboratories Inc., Montgomery, TX, USA). Briefly, serum samples and standards were added to the ELISA plates, and immunoglobulin concentrations were calculated from standard curves generated for each assay. Measurements were performed in technical triplicate, and mean values were expressed as ng/mL. Absorbance was measured using an ELISA-compatible microplate reader at the wavelength recommended by the kit manufacturer.

### 2.9 Gene expression analysis

At 42 days of age, the same two birds per replicate pen selected for carcass evaluation were used for hepatic gene-expression analysis. Immediately after euthanasia, liver samples were excised, rinsed with sterile 0.90% NaCl solution to remove residual blood, snap-frozen in liquid nitrogen, and stored at −80°C until RNA extraction. Total RNA was isolated from frozen liver tissue using a commercial total RNA extraction kit (Pars Tous Com., Mashhad, Iran) according to the manufacturer’s protocol, as described previously (Biabani et al., 2024; Mohammadizad et al., 2025). RNA concentration and purity were assessed spectrophotometrically by measuring absorbance and the A260/A280 and A260/A230 ratios, respectively, using an Epoch microplate spectrophotometer (BioTek, Winooski, VT, USA). Residual genomic DNA was removed using DNase I (Thermo Fisher Scientific, Austin, TX, USA), and complementary DNA (cDNA) was synthesized from purified RNA using a commercial cDNA synthesis kit (Pars Tous Com., Mashhad, Iran) according to the manufacturer’s instructions.

Quantitative real-time PCR (qPCR) was performed to quantify the relative hepatic mRNA expression of genes related to antioxidant defense (*NFE2L2, GPX1, SOD1*, and *CAT*) and immune regulation (*NF-κB, IL-1β, IL-6*, and *IL-10*). *GAPDH* was used as the internal reference gene. Complete forward and reverse primer sequences, amplicon lengths, and GenBank accession numbers are provided in Supplementary Table S1; the primer metadata were verified against those reported in previous broiler studies (Biabani et al., 2024; Mohammadizad et al., 2024). RT-qPCR reactions were performed with SYBR Green qPCR Master Mix (Pars Tous Com., Mashhad, Iran) in a final volume of 20 µL using an ABI 7300 Real-Time PCR System (Applied Biosystems, Foster City, CA, USA). All reactions were performed in technical triplicate. Relative gene expression was calculated using the 2^−ΔΔCt^ method, with target-gene Ct values normalized to *GAPDH* and expressed relative to those of the Control group, which served as the calibrator, following MIQE-based reporting principles.

### 2.10 Targeted ileal and cecal bacterial counts

On day 42, two birds per replicate pen, with body weights close to the pen mean, were selected for targeted intestinal bacterial enumeration. After euthanasia, the gastrointestinal tract was aseptically excised, and the ileum (from Meckel’s diverticulum to the ileocecal junction) and paired ceca (from the cecal ostia to the tips) were identified and ligated to minimize cross-contamination. Approximately 1 g of digesta from each segment was collected into sterile containers, homogenized, and serially diluted 10-fold in sterile phosphate-buffered saline. Appropriate dilutions were plated in duplicate using selective culture methods commonly applied in broiler studies (Nari et al., 2020; Nooreh et al., 2021). *Lactobacillus* spp. were enumerated on de Man, Rogosa, and Sharpe (MRS) agar after anaerobic incubation at 37–39°C for 48–72 h, whereas *Escherichia coli* colonies were enumerated on eosin methylene blue (EMB) agar after aerobic incubation at 37–39°C for 24 h. Colonies showing the expected growth characteristics on the respective selective media were counted. Duplicate-plate counts were averaged for each sample, and values from the two sampled birds were subsequently averaged to obtain a single pen-level observation for statistical analysis. Results were expressed as log10 CFU/g of digesta.

### 2.11 Tibia quality and mineral analysis

At 42 days of age, the two birds from each pen previously selected for carcass evaluation were also used for tibia sampling. After euthanasia and carcass dissection, both tibiae were carefully removed and cleaned of adhering soft tissues. The right tibia was used for morphological and mechanical evaluations. Tibia weight was recorded, whereas tibia length and diameters at the widest and narrowest points were measured using a digital caliper, as described previously (Ghasemi et al., 2020; Mohammadizad et al., 2024). Tibia breaking strength was determined using a mechanical testing instrument (Instron model 5500; Instron Corp., Canton, MA, USA), following procedures described in previous bone-quality studies. Relative tibia weight was calculated as a percentage of pre-slaughter live body weight.

The left tibia was used for ash determination and mineral analysis. Briefly, the left tibiae were crushed, defatted with petroleum ether for 24 h using Soxhlet extraction, and dried in an oven at 100°C for 24 h. The dried, fat-free tibia samples were then ashed in a muffle furnace at 600°C to determine tibia ash percentage on a dry, fat-free basis. The resulting ash was solubilized using a nitric acid–perchloric acid mixture, and the concentrations of calcium, phosphorus, magnesium, potassium, sodium, iron, zinc, manganese, and copper were determined by inductively coupled plasma optical emission spectrometry (ICP-OES). In accordance with previous studies, mineral analysis was performed using an ICP-AES system (Optima 7000 DV; PerkinElmer, Waltham, MA, USA) (Ghasemi et al., 2020; Ghasemi et al., 2023).

### 2.12 Statistical analysis

Data were analyzed according to a completely randomized design using the GLM procedure of SAS (version 9.4; SAS Institute Inc., Cary, NC, USA), with experimental treatment as the fixed effect. The pen was the experimental unit for performance variables and for all other outcomes after values from the two sampled birds within each pen were averaged; thus, individual birds were treated as subsamples. Model residuals were assessed for normality before final analysis. Percentage data, including carcass traits and relative organ weights, were subjected to arcsine square-root transformation before analysis, whereas untransformed means are presented for interpretation. Treatment means were compared using the LSMEANS option with Tukey adjustment for multiple comparisons. Results are presented as means with their SEM. Statistical significance was defined as P < 0.05.

## 3 Results

### 3.1 Growth performance and carcass traits

Table 3 summarizes growth performance during days 1–42 and carcass traits at day 42. Treatment affected body weight gain (BWG; P < 0.001), feed intake (P = 0.013), and feed efficiency (P < 0.001). The AF group had lower BWG than the Control group. Relative to AF, AF+TB increased BWG by 9.7% and feed efficiency by 6.8%, whereas AF+NZ+NS increased BWG by 6.3% and feed efficiency by 5.7%; both treatments resulted in significantly higher BWG and feed efficiency than AF (P < 0.001). Feed intake was lower in AF than in the Control group, while the supplemented AFB1-challenged groups were intermediate. AF+NZ+NS did not differ significantly from AF+TB for BWG and feed efficiency but did not consistently differ from the individual nano-mineral treatments across performance indices.

**Table 3:** Effects of nano-zinc and nano-selenium on growth performance and slaughter variables (at 42 days of age) in broiler chickens fed an AFB1-contaminated diet.

	Experimental groups^1^							
Item^2^	Control	AF	AF+TB	AF+NZ	AF+NS	AF+NZ+NS	SEM	P-value
Performance indices								
Body weight gain	2918^a^	2600^d^	2853^ab^	2695^cd^	2661^cd^	2765^bc^	28.3	<0.001
Feed intake	4934^a^	4787^b^	4916^ab^	4843^ab^	4802^ab^	4821^ab^	32.6	0.013
Feed efficiency	0.591^a^	0.543^d^	0.580^ab^	0.557^bcd^	0.554^cd^	0.574^abc^	0.0058	<0.001
Slaughter traits^2^								
Carcass	73.59^a^	71.64^b^	73.28^a^	73.02^ab^	72.56^ab^	73.18^ab^	0.371	0.013
Breast	26.09^a^	23.96^b^	25.89^a^	25.42^a^	25.30^ab^	25.98^a^	0.337	0.001
Thigh	11.36^a^	10.40^b^	11.40^ab^	10.93^ab^	10.73^ab^	11.20^ab^	0.238	0.038
Drumstick	10.45^a^	9.59^b^	10.38^ab^	10.05^ab^	9.92^ab^	10.26^ab^	0.190	0.030
Abdominal Fat	0.98^c^	1.36^a^	1.07^bc^	1.21^ab^	1.19^abc^	1.13^bc^	0.053	0.001
Liver	1.35^b^	1.58^a^	1.42^ab^	1.51^ab^	1.48^ab^	1.45^ab^	0.039	0.005
Heart	0.478	0.557	0.503	0.524	0.513	0.495	0.0187	0.091
Spleen	0.191	0.166	0.188	0.175	0.173	0.182	0.0062	0.059
Thymus	0.255^a^	0.214^b^	0.246^ab^	0.237^ab^	0.242^ab^	0.240^ab^	0.0087	0.049
Bursa	0.145^a^	0.118^b^	0.141^a^	0.133^ab^	0.127^ab^	0.139^a^	0.0047	0.003

Means within each row series followed by different lowercase letters differ significantly (*P* < 0.05).

1 Control: Basal diet without AFB1 or supplements; AF: Basal diet contaminated with AFB1 at 0.5 mg/kg; AF+TB: AF diet supplemented with a commercial toxin binder at 2 g/kg; AF+NZ: AF diet supplemented with 40 mg/kg dietary zinc from a nano-zinc source, replacing an equivalent amount of inorganic zinc (zinc sulfate); AF+NS: AF diet supplemented with 0.1 mg/kg dietary selenium from a nano-selenium source, replacing an equivalent amount of inorganic selenium (sodium selenite); AF+NZ+NS: AF diet supplemented with 40 mg/kg dietary zinc from a nano-zinc source and 0.1 mg/kg dietary selenium from a nano-selenium source.

2 Based on preslaughter body weight (%)

Treatment effects were detected for carcass yield (P = 0.013), breast yield (P = 0.001), thigh yield (P = 0.038), drumstick yield (P = 0.030), abdominal fat (P = 0.001), relative liver weight (P = 0.005), thymus weight (P = 0.049), and bursa weight (P = 0.003). Carcass yield was higher in the Control and AF+TB groups than in AF, and breast yield was higher in all groups except AF+NS than in AF (P < 0.05). Abdominal fat was lower in the Control, AF+TB, and AF+NZ+NS groups than in AF. Relative liver and thymus weights were higher in AF than in the Control group, whereas relative bursa weight was higher in the Control, AF+TB, and AF+NZ+NS groups than in AF. Relative heart (P = 0.091) and spleen (P = 0.059) weights were not significantly affected by treatment.

### 3.2 Serum antioxidant parameters, hormonal profile, and antioxidant-related gene expression

Serum antioxidant outcomes are presented in Table 4. Treatment affected TAC (P < 0.001), GPx activity (P = 0.001), MDA (P < 0.001), and GSH (P < 0.001), whereas no significant treatment effects were detected for SOD (P = 0.178) or CAT (P = 0.400) activity. Relative to AF, TAC increased by 37.6% in AF+TB and 34.5% in AF+NZ+NS; serum GSH increased by 38.6% and 35.7%, respectively, while MDA decreased by 31.0% and 32.1%, respectively. The Control group had higher GPx activity than the AF and AF+NZ groups.

**Table 4:** Effects of nano-zinc and nano-selenium on serum antioxidant status and hormone profile (at 42 days of age) in broiler chickens fed an AFB1-contaminated diet.

	Experimental groups^1^							
Item	Control	AF	AF+TB	AF+NZ	AF+NS	AF+NZ+NS	SEM	P-value
TAC, U/mL	5.02^a^	3.51^c^	4.83^ab^	4.23^b^	4.52^ab^	4.72^ab^	0.162	<0.001
GPx, U/mL	1459^a^	1126^b^	1310^ab^	1147^b^	1313^ab^	1336^ab^	51.4	0.001
SOD, U/mL	141.7	115.1	133.7	120.6	128.6	137.7	7.94	0.178
CAT, U/mL	2.59	2.14	2.47	2.39	2.42	2.60	0.162	0.400
MDA, nmol/mL	2.27^b^	3.58^a^	2.47^b^	2.92^ab^	2.73^b^	2.43^b^	0.160	<0.001
GSH, ng/mL	5.02^a^	3.50^c^	4.85^ab^	4.09^bc^	4.61^ab^	4.75^ab^	0.180	<0.001
Corticosterone, ng/mL	6.31^c^	8.81^a^	7.58^abc^	7.92^ab^	8.01^ab^	7.33^bc^	0.322	<0.001
T3, ng/mL	2.49	1.88	2.17	1.96	2.04	2.01	0.155	0.117
T4, ng/mL	10.16^a^	7.50^b^	9.60^a^	8.62^ab^	8.73^ab^	9.10^ab^	0.465	0.008

Means within each row series followed by different lowercase letters differ significantly (*P* < 0.05).

Abbreviations: TAC, total antioxidant capacity; GPx, glutathione peroxidase; SOD, superoxide dismutase; CAT, catalase; MDA, malondialdehyde; T3, triiodothyronine; T4, thyroxine.

1 Control: Basal diet without AFB1 or supplements; AF: Basal diet contaminated with AFB1 at 0.5 mg/kg; AF+TB: AF diet supplemented with a commercial toxin binder at 2 g/kg; AF+NZ: AF diet supplemented with 40 mg/kg dietary zinc from a nano-zinc source, replacing an equivalent amount of inorganic zinc (zinc sulfate); AF+NS: AF diet supplemented with 0.1 mg/kg dietary selenium from a nano-selenium source, replacing an equivalent amount of inorganic selenium (sodium selenite); AF+NZ+NS: AF diet supplemented with 40 mg/kg dietary zinc from a nano-zinc source and 0.1 mg/kg dietary selenium from a nano-selenium source.

For the hormone profile (Table 4), treatment affected corticosterone (P < 0.001) and T4 (P = 0.008), but not T3 (P = 0.117). AF+NZ+NS reduced serum corticosterone by 16.8% relative to AF and had a significantly lower concentration than AF (P < 0.05). Serum T4 was higher in the Control and AF+TB groups than in AF.

For antioxidant-related gene expression (Fig. 1), hepatic *NFE2L2* expression at 42 days of age was higher in the Control group than in all groups except AF+TB and AF+NZ+NS (P < 0.05). All supplemented groups showed higher *GPX1* expression than the AF group (P < 0.05). *GPX1* expression was also higher in the Control group than in the AF+NS group (P < 0.05). The AF+NZ+NS group showed higher *SOD1* expression than the AF group (P < 0.05). No significant treatment effect was detected for *CAT* expression (P > 0.05).

**Fig. 1:**
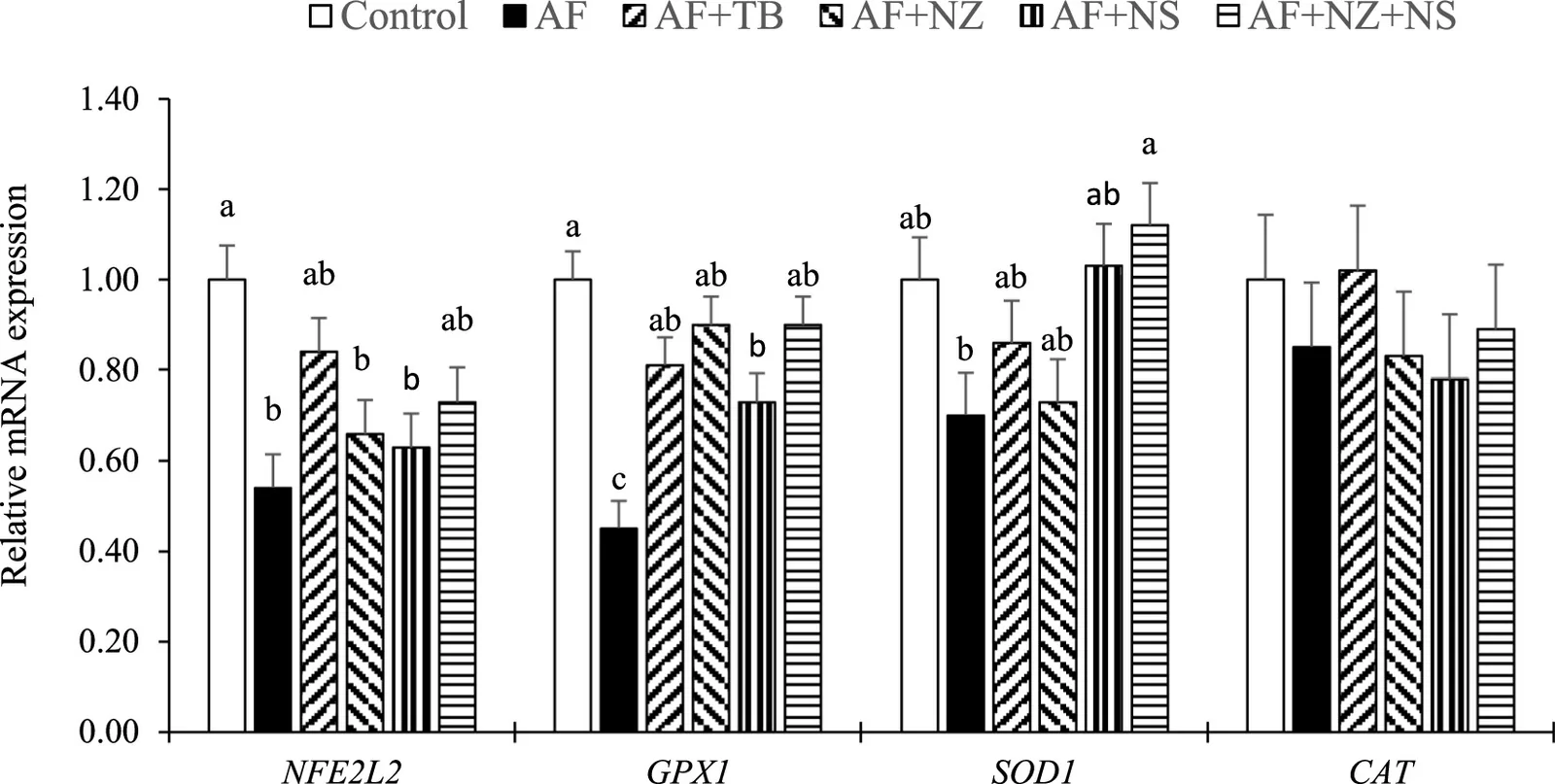
Antioxidant-related hepatic gene expression in AFB1-challenged broilers. Relative mRNA expression of nuclear factor erythroid 2-related factor 2 (*NFE2L2*), glutathione peroxidase 1 (*GPX1*), superoxide dismutase 1 (*SOD1*), and catalase (*CAT*) in 42-day-old broiler chickens fed Control or AFB1-contaminated diets with or without a toxin binder, nano-zinc, nano-selenium, or their combination. Error bars indicate SEM. Different letters within the same gene indicate differences among treatments according to Tukey’s multiple-comparison test (P < 0.05). Values are means of 6 replicate pens per treatment, with 2 chickens sampled per replicate pen. Treatment abbreviations: Control, basal diet without AFB1 or supplements; AF, basal diet contaminated with AFB1 at 0.5 mg/kg; AF+TB, AF diet supplemented with a commercial toxin binder at 2 g/kg; AF+NZ, AF diet supplemented with 40 mg/kg dietary zinc from a nano-zinc source replacing an equivalent amount of zinc sulfate; AF+NS, AF diet supplemented with 0.1 mg/kg dietary selenium from a nano-selenium source replacing an equivalent amount of sodium selenite; AF+NZ+NS, AF diet supplemented with both nano-zinc and nano-selenium.

### 3.3 Serum immunoglobulin concentrations and immune-related gene expression

Table 5 presents serum immunoglobulin concentrations on days 24 and 42. Treatment effects were detected for day-24 IgG (P < 0.001) and IgM (P = 0.006) and for day-42 IgG (P = 0.001), IgM (P = 0.006), and IgA (P = 0.002); no significant treatment effect was detected for day-24 IgA (P = 0.201). On day 24, IgG was 32.4% higher in AF+TB and 23.2% higher in AF+NZ+NS than in AF, with both groups showing significantly higher concentrations than AF (P < 0.05). On day 42, IgA was 48.6% higher in AF+TB and 40.6% higher in AF+NZ+NS than in AF, and both differences were significant. The individual nano-mineral groups showed intermediate or inconsistent responses across immunoglobulin classes and sampling days.

**Table 5:** Effects of nano-zinc and nano-selenium on serum immunoglobulin concentrations (at 24 and 42 days of age) in broiler chickens fed an AFB1-contaminated diet.

	Experimental groups^1^							
Item (ng/mL)	Control	AF	AF+TB	AF+NZ	AF+NS	AF+NZ+NS	SEM	P-value
Day 24								
IgG	1392^a^	968^c^	1282^ab^	1107^bc^	1094^bc^	1193^ab^	50.68	<0.001
IgM	174.7^a^	147.3^b^	165.8^ab^	153.3^ab^	145.4^b^	150.6^ab^	5.73	0.006
IgA	446.9	303.2	383.7	322.6	336.0	353.9	41.29	0.201
Day 42								
IgG	1192^a^	884^b^	1031^ab^	961^b^	941^b^	1055^ab^	45.80	0.001
IgM	223.5^ab^	196.1^bc^	225.8^a^	206.6^abc^	193.5^c^	209.2^abc^	6.66	0.006
IgA	572.9^a^	361.9^b^	537.8^a^	468.9^ab^	446.0^ab^	508.8^a^	34.16	0.002

Means within each row series followed by different lowercase letters differ significantly (*P* < 0.05).

1 Control: Basal diet without AFB1 or supplements; AF: Basal diet contaminated with AFB1 at 0.5 mg/kg; AF+TB: AF diet supplemented with a commercial toxin binder at 2 g/kg; AF+NZ: AF diet supplemented with 40 mg/kg dietary zinc from a nano-zinc source, replacing an equivalent amount of inorganic zinc (zinc sulfate); AF+NS: AF diet supplemented with 0.1 mg/kg dietary selenium from a nano-selenium source, replacing an equivalent amount of inorganic selenium (sodium selenite); AF+NZ+NS: AF diet supplemented with 40 mg/kg dietary zinc from a nano-zinc source and 0.1 mg/kg dietary selenium from a nano-selenium source.

Regarding immune-related gene expression (Fig. 2), *NF-κB* expression was higher in the AF and AF+NS groups than in the Control group (P < 0.05). *IL-1β* expression was lower in the Control and AF+TB groups than in the AF group (P < 0.05). All groups, except AF+NS, showed lower *IL-6* expression than the AF group (P < 0.05). *IL-6* expression in the Control group was lower than in all groups except AF+TB (P < 0.05). Conversely, *IL-10* expression was higher in the Control and AF+TB groups than in the AF group (P < 0.05). Among the supplemented groups, AF+NZ+NS showed an intermediate *IL-10* response and did not differ significantly from the other groups (P > 0.05).

**Fig. 2:**
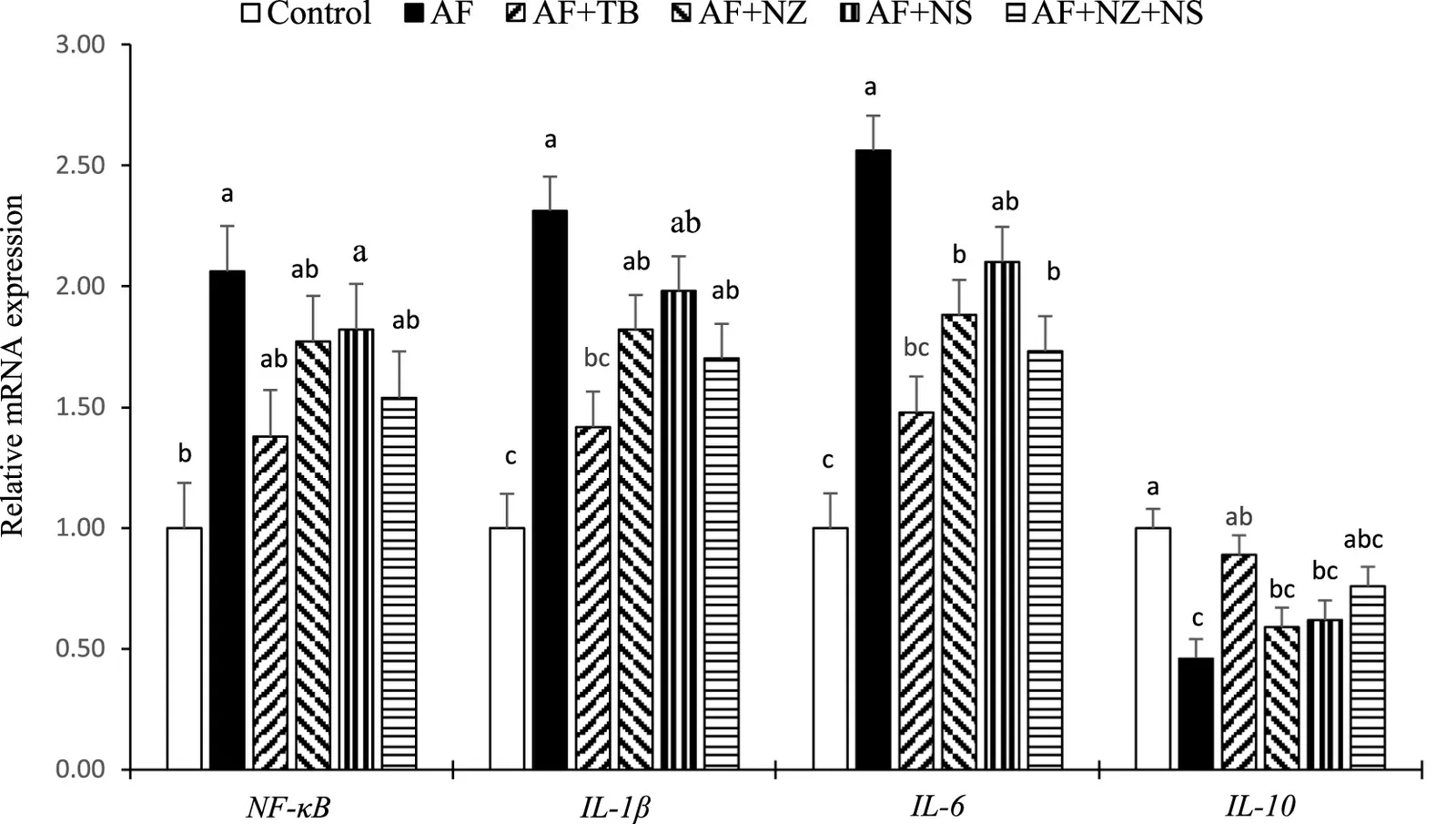
Immune-related hepatic gene expression in AFB1-challenged broilers. Relative mRNA expression of nuclear factor kappa B (*NF-κB*), interleukin-1 beta (*IL-1β*), interleukin-6 (*IL-6*), and interleukin-10 (*IL-10*) in 42-day-old broiler chickens fed Control or AFB1-contaminated diets with or without a toxin binder, nano-zinc, nano-selenium, or their combination. Error bars indicate SEM. Different letters within the same gene indicate differences among treatments according to Tukey’s multiple-comparison test (P < 0.05). Values are means of 6 replicate pens per treatment, with 2 chickens sampled per replicate pen. Treatment abbreviations: Control, basal diet without AFB1 or supplements; AF, basal diet contaminated with AFB1 at 0.5 mg/kg; AF+TB, AF diet supplemented with a commercial toxin binder at 2 g/kg; AF+NZ, AF diet supplemented with 40 mg/kg dietary zinc from a nano-zinc source replacing an equivalent amount of zinc sulfate; AF+NS, AF diet supplemented with 0.1 mg/kg dietary selenium from a nano-selenium source replacing an equivalent amount of sodium selenite; AF+NZ+NS, AF diet supplemented with both nano-zinc and nano-selenium.

### 3.4 Ileal and cecal bacterial counts

Table 6 presents targeted culture-based bacterial counts at 42 days of age. Treatment affected ileal *Lactobacillus* spp. (P = 0.029), ileal *E. coli* (P = 0.002), cecal *Lactobacillus* spp. (P < 0.001), and cecal *E. coli* (P < 0.001). In the ileum, AF+NZ and AF+NZ+NS had lower *E. coli* counts than AF and did not differ significantly from the Control group. In the cecum, AF+TB had higher *Lactobacillus* counts than AF, whereas AF+TB and AF+NZ+NS had lower *E. coli* counts than AF (P < 0.05).

**Table 6:** Effects of nano-zinc and nano-selenium on targeted culture-based bacterial counts (mean log10 CFU/g) in the ileum and cecum at 42 days in broiler chickens fed an AFB1-contaminated diet.

	Experimental groups^1^							
Item	Control	AF	AF+TB	AF+NZ	AF+NS	AF+NZ+NS	SEM	P-value
Ileum *Lactobacillus*	7.72^a^	6.60^b^	7.21^ab^	6.94^ab^	7.02^ab^	7.32^ab^	0.220	0.029
*E. coli*	6.08^b^	7.83^a^	6.84^ab^	6.54^b^	6.95^ab^	6.56^b^	0.265	0.002
Cecum *Lactobacillus*	9.10^a^	7.47^c^	8.41^ab^	7.87^bc^	7.91^bc^	8.17^bc^	0.187	<0.001
*E. coli*	7.17^c^	9.05^a^	8.02^bc^	8.45^ab^	8.72^ab^	8.10^b^	0.212	<0.001

Means within each row series followed by different lowercase letters differ significantly (*P* < 0.05).

1 Control: Basal diet without AFB1 or supplements; AF: Basal diet contaminated with AFB1 at 0.5 mg/kg; AF+TB: AF diet supplemented with a commercial toxin binder at 2 g/kg; AF+NZ: AF diet supplemented with 40 mg/kg dietary zinc from a nano-zinc source, replacing an equivalent amount of inorganic zinc (zinc sulfate); AF+NS: AF diet supplemented with 0.1 mg/kg dietary selenium from a nano-selenium source, replacing an equivalent amount of inorganic selenium (sodium selenite); AF+NZ+NS: AF diet supplemented with 40 mg/kg dietary zinc from a nano-zinc source and 0.1 mg/kg dietary selenium from a nano-selenium source.

### 3.5 Tibia quality and tibial mineral composition

The effects of dietary treatments on tibia traits and mineral composition are presented in Table 7. Treatment affected tibia length (P < 0.001), tibia weight (P = 0.023), tibia breaking strength (P = 0.026), tibia ash (P < 0.001), phosphorus (P = 0.012), and zinc (P < 0.001). Relative to AF, tibia length was 14.6% greater in AF+TB and 9.5% greater in AF+NZ+NS; both groups had significantly greater tibia length than AF. Tibial Zn concentration was 24.7% higher in AF+NZ and 22.7% higher in AF+NZ+NS than in AF, and both nano-Zn-containing treatments had significantly higher tibial Zn concentrations than AF. Tibia ash was higher in the Control and AF+TB groups than in AF, AF+NZ, and AF+NS; AF+NZ+NS was intermediate and did not differ significantly from either AF or the Control/AF+TB groups. Tibial calcium, magnesium, potassium, sodium, iron, manganese, and copper were not significantly affected by treatment (P > 0.05). Thus, nano-Zn-containing treatments were associated with higher tibial Zn concentrations than AF, whereas improvements were not observed consistently across all skeletal indices.

**Table 7:** Effect of experimental treatments on tibia quality and tibial mineral content (at 42 days of age) in broiler chickens fed an AFB1-contaminated diet.

	Experimental groups^1^							
Item^2^	Control	AF	AF+TB	AF+NZ	AF+NS	AF+NZ+NS	SEM	*P*-value
Tibia length (mm)	104.3^a^	88.1^d^	101.0^ab^	94.0^cd^	92.6^cd^	96.5^bc^	1.55	<0.001
Tibia weight (g)	14.30^a^	12.86^b^	13.92^ab^	13.53^ab^	13.13^ab^	13.57^ab^	0.297	0.023
Relative tibia bone (%)	7.27	6.57	7.16	6.90	6.74	6.97	0.196	0.156
Bone strength (N/mm²)	443.2^a^	365.5^b^	423.5^ab^	404.3^ab^	373.2^ab^	412.8^ab^	17.18	0.026
Ash (%)	51.40^a^	48.39^b^	51.51^a^	49.15^b^	48.66^b^	49.55^ab^	0.480	<0.001
Calcium (g/kg)	153.7	146.9	152.0	148.3	147.4	148.6	2.27	0.240
Phosphorus (g/kg)	86.43^a^	82.76^b^	85.82^ab^	83.97^ab^	83.32^ab^	84.33^ab^	0.758	0.012
Magnesium (g/kg)	4.53	4.28	4.58	4.37	4.24	4.43	0.202	0.805
Potassium (g/kg)	2.09	1.96	2.11	2.01	1.90	1.97	0.122	0.800
Sodium (g/kg)	5.02	4.93	5.12	4.91	4.95	5.00	0.130	0.887
Iron (mg/kg)	132.2	119.6	134.1	124.0	124.7	127.6	6.24	0.587
Zinc (mg/kg)	188.0^ab^	169.0^b^	177.3^b^	210.8^a^	174.1^b^	207.3^a^	6.60	<0.001
Manganese (mg/kg)	4.58	4.30	4.65	4.37	4.26	4.32	0.155	0.387
Copper (mg/kg)	0.818	0.776	0.794	0.780	0.768	0.785	0.0318	0.911

Means within each row followed by different lowercase superscript letters differ significantly (P < 0.05).

1 Control: Basal diet without AFB1 or supplements; AF: Basal diet contaminated with AFB1 at 0.5 mg/kg; AF+TB: AF diet supplemented with a commercial toxin binder at 2 g/kg; AF+NZ: AF diet supplemented with 40 mg/kg dietary zinc from a nano-zinc source, replacing an equivalent amount of inorganic zinc (zinc sulfate); AF+NS: AF diet supplemented with 0.1 mg/kg dietary selenium from a nano-selenium source, replacing an equivalent amount of inorganic selenium (sodium selenite); AF+NZ+NS: AF diet supplemented with 40 mg/kg dietary zinc from a nano-zinc source and 0.1 mg/kg dietary selenium from a nano-selenium source.

## 4 Discussion

The AF treatment reduced BWG, feed intake, feed efficiency, and several carcass outcomes, consistent with adverse metabolic consequences associated with AFB1-induced hepatic and systemic injury. The concurrent decline in feed intake and feed efficiency is consistent with both reduced nutrient intake and less efficient conversion of consumed feed into body weight gain. This interpretation is consistent with evidence showing that AFB1 impairs intestinal architecture, barrier integrity, and nutrient absorption (Sarker et al., 2023). Recent studies in broilers have similarly linked dietary AFB1 with growth retardation, hepatic toxin residues, oxidative injury, and altered intestinal morphology (Rao et al., 2026). In the present study, AF+TB produced the broadest and most consistent performance response, while AF+NZ+NS increased BWG by 6.3% and feed efficiency by 5.7% relative to AF and often did not differ significantly from the toxin-binder group. This pattern supports partial mitigation, but not the conclusion that the combined nano-minerals were uniformly superior to the single nano-mineral treatments. The broader broiler nutrition literature also shows that growth and carcass responses can be modified by diverse interventions, including alternative trace-mineral sources (Wijayanti et al., 2025), phytogenic supplementation (Armanini et al., 2021), and non-antibiotic antimicrobial strategies (Habib et al., 2024). These comparisons provide nutritional context rather than direct mechanistic equivalence because the interventions and challenge models differ. Accordingly, the present Zn-Se combination is best described as improving a broader set of measured responses than was consistently observed with either nano-mineral alone. Zinc contributes to Cu/Zn-SOD function, membrane stabilization, epithelial integrity, and immune competence (Akhavan-Salamat and Ghasemi, 2019; Xie et al., 2025), whereas selenium-containing selenoproteins regulate peroxide removal, redox signaling, metabolism, and inflammation (Arbabi-Motlagh et al., 2022; Chen et al., 2024a). These complementary biological roles provide a plausible basis for the broader response observed with combined nano-Zn and nano-Se; however, the present data do not establish that specific Zn-dependent or Se-dependent pathways caused the improvements in performance, carcass traits, or relative bursa weight.

The redox data indicate greater oxidative stress in AF, reflected by a pattern of lower TAC, GPx activity, GSH, *NFE2L2*, and *GPX1* responses together with higher MDA and corticosterone. These changes are consistent with bioactivation of AFB1 to reactive metabolites that promote reactive oxygen species generation, lipid peroxidation, glutathione depletion, inflammatory signaling, and suppression of *NFE2L2*-dependent antioxidant defenses (Rajput et al., 2019; Hou et al., 2022; Jobe et al., 2023). The higher MDA concentration is consistent with enhanced lipid peroxidation, whereas reductions in GSH and GPx/GPX1 are consistent with impairment of the glutathione-dependent antioxidant system (Guo et al., 2021; Lin et al., 2022; Xi et al., 2022). AF+TB and AF+NZ+NS increased TAC and GSH and reduced MDA relative to AF, while AF+NZ+NS also reduced corticosterone. For the toxin binder, reduced gastrointestinal AFB1 availability is a plausible and literature-supported mechanism (Cuccato et al., 2025). For nano-Zn and nano-Se, however, AFB1 absorption and tissue residues were not measured; therefore, their effects are more appropriately interpreted as being consistent with host redox-support responses rather than evidence of reduced toxin bioavailability. Mechanistically, Zn supports Cu/Zn-SOD, metallothionein, membrane stability, and immune-redox regulation, whereas Se is incorporated into glutathione peroxidases and thioredoxin reductases that remove downstream peroxides (Adekunle et al., 2023; Debata et al., 2023; Xie et al., 2025). A recent elemental nano-Se study also reported source-dependent improvements in growth and antioxidant-related outcomes in finishing pigs, providing cross-species mechanistic context rather than direct broiler evidence (Luo et al., 2026). The lower corticosterone concentration in AF+NZ+NS is also consistent with attenuation of the physiological stress response; however, the hormone data do not establish recovery of hepatic–endocrine function, consistent with reports that antioxidant, anti-mycotoxin, and trace-mineral interventions can modulate NFE2L2-related pathways and reduce oxidative injury in AFB1-challenged broilers (Cuccato et al., 2025; Ye et al., 2025).

AFB1 was also associated with alterations in humoral immune indices and a gene-expression profile consistent with greater pro-inflammatory signaling. This pattern agrees with reports showing that AFB1 activates NF-κB-mediated inflammatory pathways and weakens immunoglobulin responses in poultry (Hou et al., 2022; Lai et al., 2022). AF+TB produced the broadest immune response across IgM, IgA, *IL-1β, IL-6*, and *IL-10*, whereas AF+NZ+NS improved day-24 IgG and day-42 IgA and showed intermediate inflammatory-gene responses. The stronger response observed in AF+TB may reflect reduced gastrointestinal AFB1 availability and consequently lower systemic toxin exposure (Lai et al., 2022; Karimi Zandi et al., 2026); preservation of intestinal barrier function was not directly evaluated in the present study. The observed nano-mineral effects are biologically compatible with known Zn roles in lymphocyte function and redox-sensitive *NF-κB* regulation and Se roles in selenoprotein-dependent antioxidant and immune regulation (Jain et al., 2021; Elkhateeb et al., 2024; Sherif et al., 2024). Thus, complementary Zn- and Se-dependent pathways provide a plausible explanation for the immune-related responses to AF+NZ+NS, although the specific cellular mechanisms were not determined. Properties attributed to some nano-mineral preparations, including greater surface area, altered mucosal interactions, and potentially greater bioavailability, could also influence biological responses relative to conventional mineral salts (Hatab et al., 2024; Samy et al., 2025; Yang et al., 2025). Nevertheless, thymulin activity, lymphocyte proliferation, cellular immune phenotypes, and mineral bioavailability were not measured in this experiment. These mechanisms therefore remain literature-based explanations rather than demonstrated causal pathways. The combined treatment should consequently be interpreted as providing partial improvement in measured immune-related outcomes under AFB1 challenge, not complete immune restoration.

The microbiological results should likewise be interpreted narrowly. AFB1 was associated with lower *Lactobacillus* spp. counts and higher *E. coli* counts at selected intestinal sites, but the experiment quantified only these targeted groups by selective culture and did not measure microbiome diversity or community composition. This finding is consistent with evidence that AFB1 damages intestinal morphology, weakens tight junctions, alters mucosal immunity, and shifts the microbiota toward opportunistic pathogens (Hernández-Ramírez et al., 2020; Li et al., 2024; Choi et al., 2025). AF+TB increased cecal *Lactobacillus* spp. counts and reduced cecal *E. coli* counts relative to AF, consistent with reports showing that mycotoxin binders and detoxifying agents can mitigate AFB1-induced barrier damage, microbial imbalance, and intestinal inflammation (Tavangar et al., 2021; Karimi Zandi et al., 2026). Nano-Zn-containing treatments also reduced ileal *E. coli* counts and AF+NZ+NS reduced cecal *E. coli*. These findings are consistent with published antibacterial effects of ZnO nanoparticles in broilers (Bahadur et al., 2024; Yusof et al., 2023) and with the broader antimicrobial activity reported for engineered nanoparticles (Usman et al., 2025), but they do not establish restoration of overall microbial balance. Potential mechanisms such as direct bacterial-membrane interaction, Zn^2+^ release, and altered redox conditions (Opoola et al., 2024; Xie et al., 2025), or Se-mediated mucosal protection (Chen et al., 2024b) remain hypotheses because they were not directly evaluated in this study. Accordingly, the present data support only treatment-associated changes in selected culture-based bacterial counts.

The skeletal findings extend the physiological assessment of aflatoxicosis. AF reduced tibia length, weight, strength, ash, and phosphorus, consistent with reports that AFB1 can impair mineral utilization and bone microarchitecture (Paneru et al., 2024). The improvement observed in AF+TB supports this interpretation, as toxin binders can reduce gastrointestinal AFB1 bioavailability and thereby potentially help preserve mineral metabolism, gut function, and skeletal development (Mohammadi et al., 2025; Karimi Zandi et al., 2026). Nano-Zn-containing diets markedly increased tibial Zn concentration, with AF+NZ and AF+NZ+NS showing 24.7% and 22.7% higher values, respectively, than AF. This increase is biologically compatible with known Zn-dependent processes in bone, including osteoblast activity, alkaline phosphatase function, collagen synthesis, protein metabolism, and matrix mineralization (Wang et al., 2019; Vieira et al., 2020). The greater tibia length in AF+NZ+NS may be compatible with improved Zn availability and mineral utilization, but it cannot be attributed directly to a demonstrated osteoblast or collagen pathway. Previous studies have proposed that characteristics of nano-Zn sources, including small particle size and high surface area, may influence intestinal availability; accordingly, reports that nano-zinc or highly bioavailable zinc sources improve tibial mineralization, bone strength, mineral retention, and intestinal absorptive capacity in broilers (De Grande et al., 2020; Samy et al., 2025; Saber and Alian, 2026). However, Zn absorption and bioavailability were not directly measured in the present study. Because AF+NZ+NS did not fully restore all tibial indices, its effect should be interpreted as partial mitigation rather than complete recovery, with the clearest nano-Zn-associated response being the higher tibial Zn concentration.

Several limitations should temper the interpretation of these findings. First, no AFB1-free nano-Zn or nano-Se treatment groups were included, so the potential benefits or adverse effects of the nano-minerals under non-challenged conditions cannot be separated from their effects under AFB1 challenge. Second, intestinal assessment was restricted to selective culture-based counts of presumptive *Lactobacillus* spp. and *E. coli*; microbiome diversity, barrier permeability, and microbial metabolites were not measured. Third, AFB1 absorption, circulating and tissue AFB1 residues, and *in vivo* toxin binding were not measured in the nano-mineral treatments, precluding any conclusion that nano-Zn or nano-Se reduced AFB1 bioavailability.

## 5 Conclusions

Dietary AFB1 impaired broiler growth, carcass traits, antioxidant and immune responses, selected culture-based intestinal bacterial counts, and tibial development during the 42-day challenge period. The toxin binder provided the broadest and most consistent protection. Combined partial replacement with nano-Zn and nano-Se improved BWG, feed efficiency, several redox and immunoglobulin indices, selected bacterial counts, tibial length, and tibial Zn concentration, and these outcomes often did not differ significantly from those observed with the toxin binder. However, the combined treatment was not uniformly superior to the single nano-mineral treatments. These findings warrant further controlled studies incorporating broader intestinal phenotyping and toxin-residue measurements before the combined nano-mineral strategy can be generalized beyond the experimental conditions evaluated here.

## Funding

This research did not receive any specific grant from funding agencies in the public, commercial, or not-for-profit sectors.

## Ethics declaration

This study was conducted in accordance with the ARRIVE (Animal Research: Reporting of In Vivo Experiments) guidelines. This study was approved by the Animal Ethics Committee of Ilam University. (Approval No. IR.ILAM.REC.1405.003).

## CRediT authorship contribution statement

**Fouad Kadhim Mohsin:** Writing – original draft, Investigation, Data curation. **Kamran Taherpour:** Writing – review & editing, Supervision, Project administration, Methodology, Conceptualization. **Hossein Ali Ghasemi:** Writing – review & editing, Supervision, Methodology, Conceptualization, Formal analysis. **Hassan Shirzadi:** Writing – review & editing, Resources, Methodology. **Fatemeh Tavakolinasab:** Writing – review & editing, Investigation, Formal analysis.

## Declaration of competing interest

The authors declare that they have no known competing financial interests or personal relationships that could have appeared to influence the work reported in this paper.

## Data Availability

Data supporting the findings of this study are available from the corresponding author upon reasonable request.
