# Supplementary material for: Protective effects of nano-zinc and nano-selenium compared with a toxin binder in broiler chickens fed aflatoxin B1-contaminated diets through antioxidant, immune, and skeletal responses
Source: Vet Anim Sci. 2026 Oct 6;34:100888. doi: 10.1016/j.vas.2026.100888 (PMC13677327; doi:10.1016/j.vas.2026.100888)
Supplement: Supplementary file 1 [file mmc1.docx]

**Table S1. Gene-specific primers for quantitative real-time PCR.**

| **Gene** | **Primer sequence 5^’^−3^’^** | **GenBank number** |
| --- | --- | --- |
| *NF-κB* | F: AGAAAAGCTGGGTCTTGGCA | NM_205134 |
|  | R: CCATCTGTGTCAAAGCAGCG |  |
| *IL-6* | F: TTCAGCAATGGCAACAGCAATG | NM_204628.2 |
|  | R: ATAGCAACAAGCGTCGTATTTCAAC |  |
| *IL-10* | F: GCTCTCACACCGCCTTGC | NM_001004414.4 |
|  | R: ACTGCTTAACTGCTATCACTAACTCTC |  |
| *IL-1β* | F: TCTTCTACCGCCTGGACAGC | XM_046931582.1 |
|  | R: TAGGTGGCGATGTTGACCTG |  |
| *NFE2L2* | F: GGCTTCTCCAGCTGCATTTC | XM_046921130.1 |
|  | R: TACTTCAGCCAGGTTGTCGTT |  |
| *GPX1* | F: CCATGTTCGAGAAGTGCGAGG | NM_001277853.3 |
|  | R: TGTACTGCGGGTTGGTCATCA |  |
| *SOD1* | F: AATCTCATTACTACTCTGCGTTCTTG | NM_205064.2 |
|  | R: CCCAATCAACCATCTTCCATTACAC |  |
| *CAT* | F: GTTGGCGGTAGGAGTCTGGTCT | NM_001031215.1 |
|  | R: GTGGTCAAGGCATCTGGCTTCTG |  |
| *GAPDH* | F: CAGAACATCATCCCAGCGTCCAC | NM_204305.2 |
|  | R: CGGCAGGTCAGGTCAACAACAG |  |

Abbreviations: *NF-kB,* nuclear factor kappa B; *IL-1β*, interleukin 1 beta; *IL-6*, interleukin 6; *IL-10*, interleukin 10; *NFE2L2*, nuclear factor erythroid 2 like 2; *GPX1*, glutathione peroxidase 1; *SOD1*, superoxide dismutase 1; CAT, catalase; *GAPDH*, glyceraldehyde-3-phosphate dehydrogenase.

F = forward primer; R = reverse primer.
